# Efficacy, Treatment Characteristics, and Biopsychological Mechanisms of Music-Listening Interventions in Reducing Pain (MINTREP): Study Protocol of a Three-Armed Pilot Randomized Controlled Trial

**DOI:** 10.3389/fpsyt.2020.518316

**Published:** 2020-11-04

**Authors:** Anja C. Feneberg, Mattes B. Kappert, Rosa M. Maidhof, Bettina K. Doering, Dieter Olbrich, Urs M. Nater

**Affiliations:** ^1^Clinical Psychology of Adulthood, Department of Clinical and Health Psychology, Faculty of Psychology, University of Vienna, Vienna, Austria; ^2^Clinical Biopsychology, Department of Psychology, University of Marburg, Marburg, Germany; ^3^Division of Clinical and Biological Psychology, Department of Psychology, Catholic University Eichstätt-Ingolstadt, Eichstätt, Germany; ^4^Center for Psychosomatic Rehabilitation, Klinik Lipperland, Bad Salzuflen, Germany

**Keywords:** autonomic nervous system, cold pressor test, music, music-induced analgesia, music intervention, pain management, stress reduction

## Abstract

**Background:** Pain can severely compromise a person's overall health and well-being. Music-listening interventions have been shown to alleviate perceived pain and to modulate the body's stress-sensitive systems. Despite the growing evidence of pain- and stress-reducing effects of music-listening interventions from experimental and clinical research, current findings on music-induced analgesia are inconclusive regarding the role of specific treatment characteristics and the biopsychological mechanisms underlying these effects.

**Objective:** The overall aim of this pilot randomized controlled trial is to test and compare the differential effects of frequency-modulated and unmodulated music (both researcher-selected) on experimentally induced perception of acute pain and to test the efficacy of the interventions in reducing biological and subjective stress levels. Moreover, these two interventions will be compared to a third condition, in which participants listen to self-selected unmodulated music.

**Methods and Analysis:** A total of 90 healthy participants will be randomly allocated to one of the three music-listening intervention groups. Each intervention encompasses 10 sessions of music listening in our laboratory. Frequency-modulation will involve stepwise filtering of frequencies in the audible range of 50–4,000 Hz. Acute pain will be induced via the cold pressor test. Primary (i.e., pain tolerance, perceived pain intensity) and secondary (i.e., heart rate variability, electrodermal activity, hair cortisol, subjective stress) outcomes will be measured at baseline, post, and follow-up. In addition, intermittent measurements as well as a follow-up assessment and a range of tertiary measures (e.g., music-induced emotions) are included.

**Discussion:** This is the first study to systematically test and compare the effects of music frequencies along with the control over music selection, both of which qualify as central treatment characteristics of music-listening interventions. Results will be highly informative for the design of subsequent large-scale clinical trials and provide valuable conclusions for the implementation of music-listening interventions for the reduction of perceived pain.

**Clinical Trial Registration:** Clinical Trials Database of the U.S. National Library of Medicine: Identifier NCT02991014.

## Introduction

The experience of pain is a multifaceted and highly individual phenomenon that involves sensory, affective, cognitive, social, and biological components. Perceived pain can cause serious disruptions in daily functioning and often compromises an individual's well-being and quality of life (1, 2). While acute pain is defined as a “sensory and emotional experience associated with actual or potential tissue damage, or described in terms of such damage” that is mostly unpleasant, though temporary (3), chronic pain typically lasts several months or even years, occurs with or without an underlying somatic cause, and affects a large proportion of our society (4). For instance, pain conditions including low back pain and migraine are amongst the leading causes of disability and disease burden worldwide (4). In addition to the individual suffering, the treatment of chronic pain is associated with tremendously high direct and indirect costs for society as a whole (5). For these reasons, chronic pain is considered a global health challenge that needs to be treated by affordable, effective, and well-accepted interventions (6–8).

Over the last decades, music as an adjuvant treatment for the management of both acute and chronic pain has received growing interest in clinical practice and scientific research [e.g., (9, 10)]. “Music-induced analgesia” offers several advantages, since music is cost-effective, non-invasive, easy to (self-)administer and does not have the drawback of severe side effects as compared to most pharmacological treatments (11, 12). A vast body of evidence supports the pain-reducing effects of music in diverse conditions, including surgical and chronic pain. While surgical (i.e., postoperative) pain can be considered (mostly) transient and functional to a certain degree (e.g., by causing the individual to protect affected body parts), chronic pain is not (or no longer) related to actual tissue damage, exceeds the healing period and is therefore considered dysfunctional (13). In a recent meta-analysis by Garza-Villareal et al. (14) including 14 randomized controlled trials and a total of 1,178 chronic pain patients, music interventions (mostly listening to recorded music) have been shown to reduce self-rated pain and comorbid psychological symptoms with moderate to large effect sizes. Two further meta-analyses of music-based interventions for cancer patients also found effects on pain ratings in the moderate to high range and additional positive effects on symptoms of anxiety, depression, and autonomic functioning (15, 16). Moreover, various meta-analyses summarizing the empirical evidence in the context of pre-, intra-, and post-operative periods of surgery have documented beneficial effects of music on ratings of pain and anxiety, as well as a reduction in anesthetic medication intake during postoperative recovery (12, 17, 18). In sum, the overall findings support the beneficial role of music-based interventions for the reduction of pain in diverse settings and patient populations. Nevertheless, it has been consistently emphasized that methodological shortcomings and a large study heterogeneity leave many questions unanswered [e.g., (11, 12, 14)]. Therefore, despite a growing body of promising empirical evidence, the literature on music-induced analgesia is still inconclusive with regard to the optimal treatment characteristics as well as the biopsychological mechanisms underlying the effects of music listening on pain perception [see also (10)].

In addition to clinical studies with patient populations, laboratory-based studies with healthy participants have elucidated several important moderating and mediating factors with respect to the effects of music listening on acute pain perception (outlined below). Although the evidence from these studies is certainly restricted in terms of generalizability to chronic pain patients, such approaches allow standardizing the magnitude of pain stimulation as well as keeping confounding variables to a minimum (19), which is of great advantage when investigating new treatment characteristics and mechanisms of action. For these reasons, we will conduct a pilot study that includes an experimental induction of acute pain in healthy participants in order to determine the differential efficacy of three music-listening interventions in reducing perceived pain and to examine the biopsychological mechanisms underlying the (potential) intervention effects.

Although various theories have been proposed with respect to the *psychological and biological mechanisms underlying music-induced analgesia*, the mediating processes are still not yet very well-understood (9, 10). The processing of pain is modulated via descending pathways, neurotransmitter systems, and neuronal/synaptic activity changes involving cortical and subcortical brain regions, the brainstem, and the spinal cord [see (20) for review]. Psychological processes, especially changes in attention and emotional state, are suggested to influence the processing of pain (21, 22). In this context, many researchers emphasize the distracting abilities of music and posit that music binds cognitive capacities, diverts the listener's attention, and consequently inhibits pain sensations [e.g., (23–25)]. In addition, emotional engagement with music might explain its pain-reducing effects. Particularly pleasurable music has been shown to induce positive emotions (e.g., joy, pleasure) in the listener and to modulate mood states (e.g., enhance feelings of relaxation, decrease feelings of anxiety) (26–28), which are assumed to be associated with a downregulation of pain experiences (29–31). In addition, research in the fields of neuroscience and psychoneuroendocrinology has revealed that music affects a multitude of cortical und subcortical areas in the brain, many of which are also involved in the processing of pain, indicating that music-induced analgesia involves the descending pain modulation pathway (9, 32). For example, previous experimental studies applying imaging techniques and pharmacological manipulations (33–35) have substantiated the idea that pleasurable music affects dopaminergic and endogenous opioid pathways that are associated with the brain's reward system. The release of dopamine and endogenous opioids in response to pleasurable music could therefore qualify as a possible biological pathway that leads to pain relief [see also (36)].

Furthermore, a biopsychological mediation model has been advocated that proposes that the autonomic nervous system (ANS) and subjective stress mediate the effects of music on pain perception (37, 38). In line with this idea, four recent systematic reviews and meta-analyses have underlined the effectiveness of music in decreasing biological and subjective markers of stress (39–42). The ANS is responsible for rapidly adapting the individual to internal and external threats via a coordinated interplay of its sympathetic and parasympathetic branch (43). Consequently, the ANS is involved in the body's processing of and response to pain, which is reflected by changes in biomarkers of the ANS such as heart rate, blood pressure, and respiration rate (44–46). Particularly heart rate variability and skin conductance are of special interest, since these indicators allow a relatively fine-grained interpretation of sympathetic and parasympathetic activity (47–49). Interestingly, the ANS is also highly sensitive to musical stimulation (50, 51). A prominent model explaining the effects of music on ANS suggests that music modulates activity in the limbic and paralimbic regions including the hippocampus and the amygdala, which are also involved in the regulation of ANS activity and the processing of pain (9, 52). To the best of our knowledge, only few studies have investigated the effects of music on autonomic functioning in the context of pain [e.g., (53–56)]. In addition, we are not aware of any experimental or clinical study that has explicitly aimed at testing changes in ANS activity as a potential mediator between music listening and pain perception. One exception to this is a previous ambulatory assessment study from our own lab, in which women diagnosed with fibromyalgia (i.e., a condition characterized by chronic widespread pain) reported on experiences of pain, stress, and their natural (i.e., experimentally not manipulated) music listening behavior multiple times per day over a duration of 14 days (38). The findings indicated that control over pain was significantly enhanced by music listening, but no effects on biological or subjective levels of stress were found [in contrast to similar studies in healthy subjects, e.g., (57)]. Therefore, the authors abstained from conducting a mediation analysis. It is noteworthy that observational studies conducted in patients' everyday life are of high value with regard to ecological validity (58); however, many parameters in daily life research cannot be controlled for, particularly in patient populations, and might have masked effects of music on ANS activity. This, again, underlines the necessity for controlled laboratory-based experimental studies with acute pain induction in healthy participants in order to unravel mechanisms of action underlying the effects of music listening on pain perception.

Next, we argue that *structural elements of music* and *choice over musical selection* are two pivotal treatment characteristics in music-listening interventions that lack a systematic investigation in previous studies (10, 59). In a recent meta-analysis by Martin-Saavedra et al. (60), the common neglect of reporting musical characteristics (e.g., tempo, instrumentation, presence of lyrics) in previous randomized controlled trials investigating music for pain management was criticized, since no clear conclusions can be drawn based on the current literature. In this regard, in their analyses of three experimental pain studies using participants' self-selected music, Knox et al. (61) found that timbral and tonal aspects were significantly correlated with measures of experimentally induced pain perception, indicating that structural elements of music might differentially moderate the effects of music on pain reduction.

In contrast to the relative lack of conclusive findings in the field of pain management, musical characteristics such as sound intensity, tempo, timbre, and arousal level have been shown to affect ANS activity in a plethora of studies (62–68). However, only recently, audio frequencies, which constitute another inherent feature of music, have been brought into focus of scientific investigations (69–72). From a technical perspective, the term frequency describes the number of oscillations per time unit, i.e., for audio frequency, it is the number of vibrations per second that determines the pitch of a sound and is measured in Hertz (Hz). Music can be defined as a combination of a fundamental frequency and multiple partial overtones, which are suggested to be translated from the cochlea into neural activity in a first step, followed by a pre-processing in the auditory brainstem, and are then analyzed in the auditory cortex and other brain regions (52). Nowadays, diverse commercial and free-to-listen compositions with frequency-modulated music are available that are claimed to exert a positive influence on the cognitive, emotional, social, and physiological domains of their consumers. Typically, these diverse programmes are based on different ideas about which frequencies might be particularly beneficial or detrimental to the human body and brain, with each programme thus justifying its specific approach of frequency modulation (e.g., amplification or filtering of certain frequencies) (73). Besides anecdotal evidence on the potential benefits of these methods, scientific research within this field is just in its infancy. For example, in the study by Nakajima et al. (71), 12 healthy women underwent a stress-inducing procedure three times in a row and listened to one of three versions of the Horn Concerto in E-flat major, K.417 by Mozart, afterwards. The respective conditions comprised the music piece modulated in the high-frequency spectrum (equal of above 3.5 kHz), in the low-frequency spectrum (below 0.5 Hz), or not modulated at all. Heart rate variability (HRV) was measured as dependent variable indicating autonomic recovery during music listening. Results indicated that particularly the modulation of high-frequency components was more effective in supporting autonomic recovery compared to the other conditions. In another study, Akimoto et al. (69) tested so-called “solfeggio frequency music.” In their study, nine participants listened to 5 min of “regular” relaxing piano music, i.e., tuned to the international standard reference tone of 440 Hz, on 1 day, and to the same piece tuned to a reference tone of 444 Hz (which results in the inclusion of 528 Hz), on a different day. Biomarkers of the ANS were assessed during and after exposure to music. Results indicated subtle differences in HRV and significant reductions in negative mood states in the 444 Hz (528 Hz), but not in the 440 Hz music condition. Thus, findings from both studies support the notion that specific frequency components in music might be capable of differentially modulating the activity of the ANS and psychological outcomes. Certainly, it is necessary to interpret the data with caution and to recognize their limited generalizability, particularly since both studies focused on short-term effects of frequency-modulated music within a single session and a small number of participants.

In addition to these experimental studies, recent investigations applied a music intervention labeled “Audiovisuelle Wahrnehmungsförderung” (AVWF®)^1^, that involves a repeated exposure to frequency-modulated music, in a clinical setting (74, 75). The authors found beneficial effects of this frequency-modulated music intervention on HRV (74) and on the cortisol-awakening response (i.e., indicator of the endocrine stress system) in psychosomatic inpatients (75). These findings, though preliminary, could indicate that the AVWF method might have a beneficial impact on biological indicators of stress in patients suffering from chronic conditions (74, 75). However, patients were not randomly assigned to treatment and control groups in the study. Thus, it remains unclear whether improvements in the variables of interest were actually caused by the frequency modulation in the music, since the mere act of music listening or other confounding aspects (e.g., selection and performance bias) cannot be ruled out based on the current findings (74, 75). Consequently, in line with the evidence reviewed above, it would be a critical endeavor to test if the beneficial impact of music on pain perception can actually be enhanced by a modulation of certain frequencies in music. Only a randomized controlled trial that compares frequency-modulated music with the same music in an unmodulated version and in which both participants and examiners are blinded with regard to frequency modulation allows drawing firm conclusions.

Furthermore, previous research is limited with regard to systematic comparisons of the effects of control over choice of music selection, i.e., researcher- vs. participants' self-selected music. Self-selected music might increase feelings of emotional and cognitive involvement during music listening (31, 55, 76). Self-selected music in contrast to pre-selected music is assumed to better capture the listener's personal preferences and is therefore thought to be associated with a higher liking of and familiarity with the music, as well as an increased sense of control, all of which have been related to pain-reducing effects (29, 31, 77). However, in some settings (e.g., hospital, rehabilitation), it might be more practical to make use of predetermined music selections. Therefore, some researchers have opted for pre-selected (i.e., researcher-selected) music pieces in the context of pain management such as slow, classical music, since this is believed to be perceived as relaxing and pleasant by most individuals [e.g., (78)]. Others offered participants to choose pieces from a variety of musical genres in order to permit some degree of personal preference [e.g., (79, 80), see also (81)]. Overall, self-selected music has been shown to be superior to music chosen by researchers/clinical staff for pain alleviation in patients with chronic pain conditions (14) and to be superior to other distracting and emotionally engaging stimuli in experimental studies with healthy participants (24).

A final shortcoming in previous research concerns the fact that the existent body of studies is inconclusive with respect to the *stability of these effects* (14). In previous laboratory-based studies, experimental pain induction and music listening are typically administered concurrently and in one session only [e.g., (31, 55, 82)]. Although this is a valuable approach for investigating the short-term impact of music on pain perception when processed in parallel, these results are of limited validity with regard to longer-lasting benefits of music for pain management. Knowledge on the required length and amount of an intervention is necessary in order to optimally balance spending of resources (e.g., temporal, financial) and desired health outcomes. In the context of music interventions for pain management, however, previous studies are characterized by a remarkable variation in length and frequency of music-listening sessions and whether pain-reducing effects of music-based interventions last over several weeks or even months have been examined only rarely. For example, Finlay (83) found short-term, but no long-term or cumulative effects of music listening on perceived pain intensity and unpleasantness in chronic pain patients, whereas other research groups found a steady increase in music-induced analgesia over 2 (78) and 4 (84) weeks, respectively. However, none of these studies included a follow-up assessment to test whether the beneficial effects of music on pain perception lasted even after cessation of the intervention period. In order to close this research gap, we chose a long duration and high number of music-listening sessions in addition to the inclusion of a (1 month) follow-up assessment in order to be able to investigate the potential intermediate stability of the effects of music listening on measures of pain perception.

## Study Aims and Hypotheses

This study addresses a number of open research questions with regard to the overall efficacy, role of specific treatment characteristics and biopsychological mechanisms of music-listening interventions in reducing perceived pain.

First, we will investigate the pain-reducing effects of frequency-modulated vs. unmodulated music, which are both researcher-selected, within a randomized controlled, laboratory-based, double-blind trial. Additionally, we include a third study arm, in which participants will listen to their self-selected (unmodulated) music. Since all study procedures (e.g., duration and number of sessions and measurements) will be conducted in parallel to the researcher-selected music-listening interventions, we will be able to directly compare the differential effects of all three music-listening interventions on measures of pain perception. Furthermore, besides measures of pain perception, we will investigate the effects of these three music-listening conditions on biomarkers of the ANS and other stress-related biological and subjective markers. In addition, we will test whether changes in these markers of stress mediate the effects of the music-listening interventions on perceived pain. We will apply a laboratory-based, experimental paradigm for the induction of acute pain (i.e., cold pressor test) at baseline, post, and follow-up, as well as intermittently during the intervention period.

Considering the complexity in design, assessments, and procedure and the relative lack of conclusive previous findings, we consider the current study a preliminary, though extensive, pilot trial. The results of this pilot randomized controlled trial (pilot-RCT) will be highly informative for the design and evaluation of subsequent large-scale trials.

This pilot-RCT targets the following main hypotheses:

1. Listening to researcher-selected frequency-modulated music and self-selected unmodulated music will result in stronger increases in pain tolerance and stronger decreases in perceived pain intensity from baseline to post-intervention compared to listening to researcher-selected unmodulated music.
2. Stronger decreases in biological and subjective markers of stress from baseline to post-intervention are expected in the researcher-selected frequency-modulated music and self-selected unmodulated music-listening conditions than in the researcher-selected unmodulated music-listening condition.
  Since there is no previous literature indicating a superiority of researcher-selected frequency-modulated or self-selected unmodulated music, we will test these comparisons in a two-sided manner.
3. Changes in pain tolerance and perceived pain intensity will be mediated by changes in biomarkers of the ANS and subjective stress.

In addition to these main hypotheses, we will examine whether reductions in measures of pain perception and markers of stress persist until the follow-up assessment (4 weeks after the post-assessment) aiming at investigating the potential intermediate stability of the effects.

Moreover, we plan to conduct additional exploratory analyses in order to unravel further health benefits as well as mediating and moderating factors in association with the music-listening interventions. For example, previous music research indicates that music interventions might improve sleep quality (85), reduce fatigue (86, 87), and enhance mood (88). Moreover, empirical evidence underlines the role of music-induced emotions and memories (26, 89), perceived musical valence and arousal (38, 57) as well as liking of and familiarity with the music (31, 90, 91) among other music-related aspects for the effects of music listening on perceived pain. Moreover, person-specific characteristics such as cognitive style of music listening (92, 93) and music-related mood regulation strategies (94) have been suggested to influence measures of pain perception and markers of stress (82, 95). Consequently, the current study aims at investigating following additional questions: are the expected pain- and stress-reducing effects stable after cessation of the interventions? Do the interventions benefit further health-related parameters, such as sleep and fatigue? What roles do music-induced perceptions (i.e., emotions, memories, chills), perceived music attributes (valence, arousal), changes in mood states, as well as liking of and familiarity with the music play in relation to measures of pain perception and markers of stress? Do the habitual cognitive style of music listening and music-related mood regulation habits moderate the effects of the music-listening interventions on measures of pain perception and markers of stress? In order to investigate these additional questions, we will assess a comprehensive set of tertiary variables.

## Methods

### Study Design

**Figure 1:**
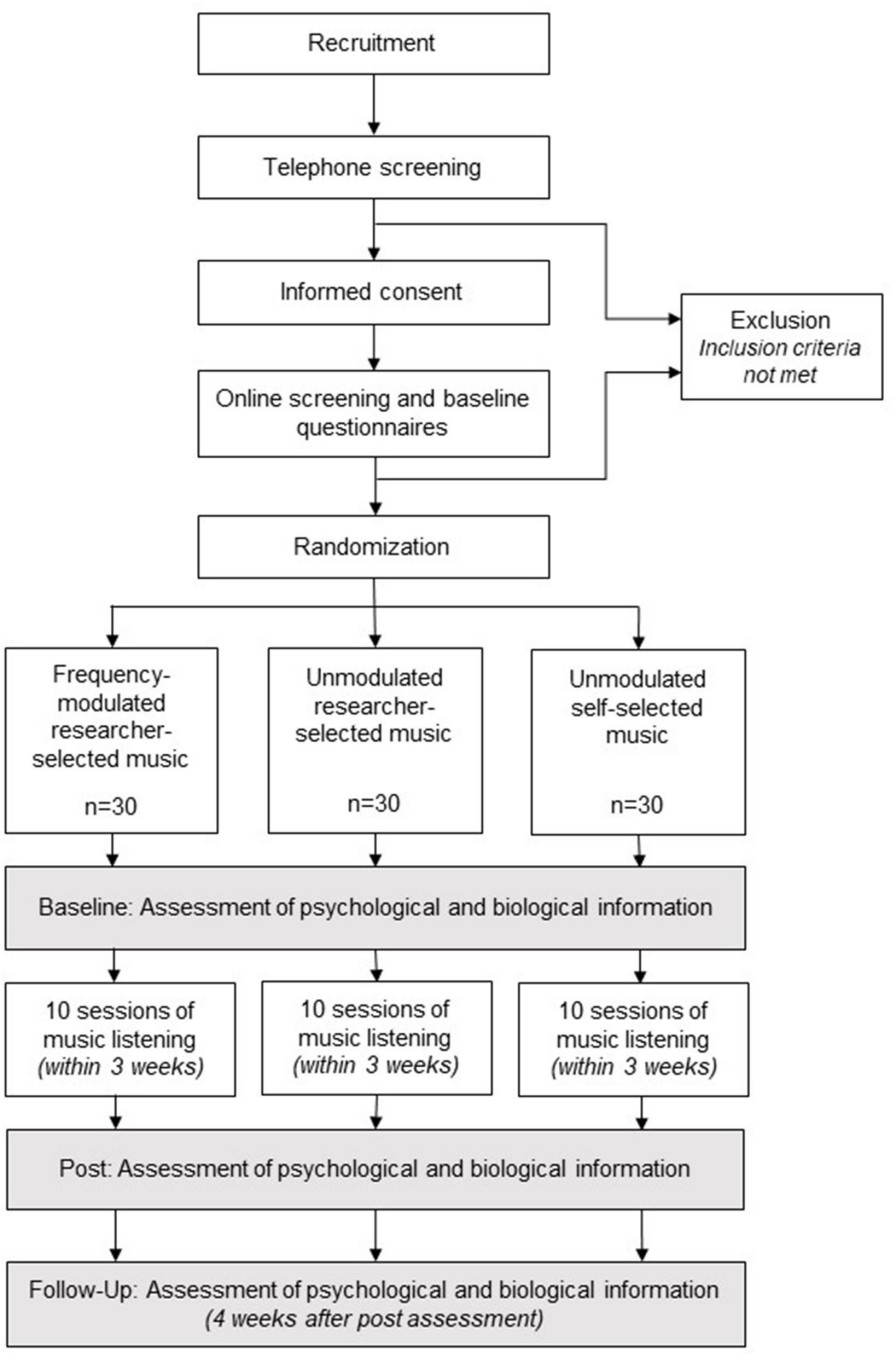
Study flow diagram.

The study is a laboratory-based, (double-)blind, randomized controlled trial with three parallel arms: frequency-modulated researcher-selected music, unmodulated researcher-selected music and unmodulated self-selected music. Double blinding with respect to frequency modulation will be achieved in the researcher-selected music conditions, and participant blinding will be ensured regarding frequency modulation within the self-selected music condition. Figure 1 displays the study flow chart.

Overall, 90 participants (30 participants per intervention condition) will attend a baseline assessment, followed by 10 sessions of music listening conducted within 3 consecutive weeks. Finally, post and follow-up assessments will be conducted. We do not anticipate protocol modifications. Nevertheless, if any trial modifications should be considered necessary, all changes in design, measures, or eligibility criteria will be recorded in the online protocol registration entry and will be included in the final manuscript for journal submission.

Following the study preparation phase (April–November 2016), recruitment and testing started in December 2016 in Marburg, Germany. Due to the move of our lab from Marburg, Germany, to Vienna, Austria, recruitment and testing needed to be paused as of January 2018 for 9 months. Testing continued in October 2018 and is presumed to be accomplished in December 2022.

### Study Setting and Procedure

All 13 appointments (baseline, 10 music-listening sessions, post-intervention assessment, follow-up assessment) will be held in our laboratory. Since previous research indicates that chronobiological rhythms influence perceived pain and stress parameters (96, 97), the appointments will be scheduled exclusively between 12 and 6 p.m. The 10 music-listening sessions (intervention period) will be scheduled within 3 consecutive weeks. Baseline and post-assessments will be held as closely in time as possible to the first and last music-listening session, respectively. Some degree of variability between participants will be accepted in order to better enable participants to fit the large number of appointments into their daily schedules.

After a telephone-based screening and upon inclusion, participants will be scheduled a baseline appointment and receive an email with instructions that they are asked to comply with in order to prevent any interference with behavioral and physiological measures (see Box 1).

Box 1 **Instructions for study appointments**

- No intake of analgesic medication on days of study appointments
- No use of body lotion or other fatty agents in the chest area (before the appointment) to minimize artifacts in ECG recordings on days of study appointments
- No consumption of alcoholic and/or energizing beverages or food, no smoking, no excessive exercise or relaxation techniques (i.e., meditation, yoga) for at least 1 hour prior to study appointments
- Wearing of comfortable clothing during appointments.

Participants allocated to the self-selected music condition will also receive an email attachment instructing them to compile 120 min of their favorite music pieces. This self-selected music compilation should be brought to the first baseline assessment on a portable device and handed to the examiner. At the end of the baseline appointment, the examiner and participant arrange further appointments for the subsequent 10 sessions of music listening within the next 3 consecutive weeks.

**Figure 2:**
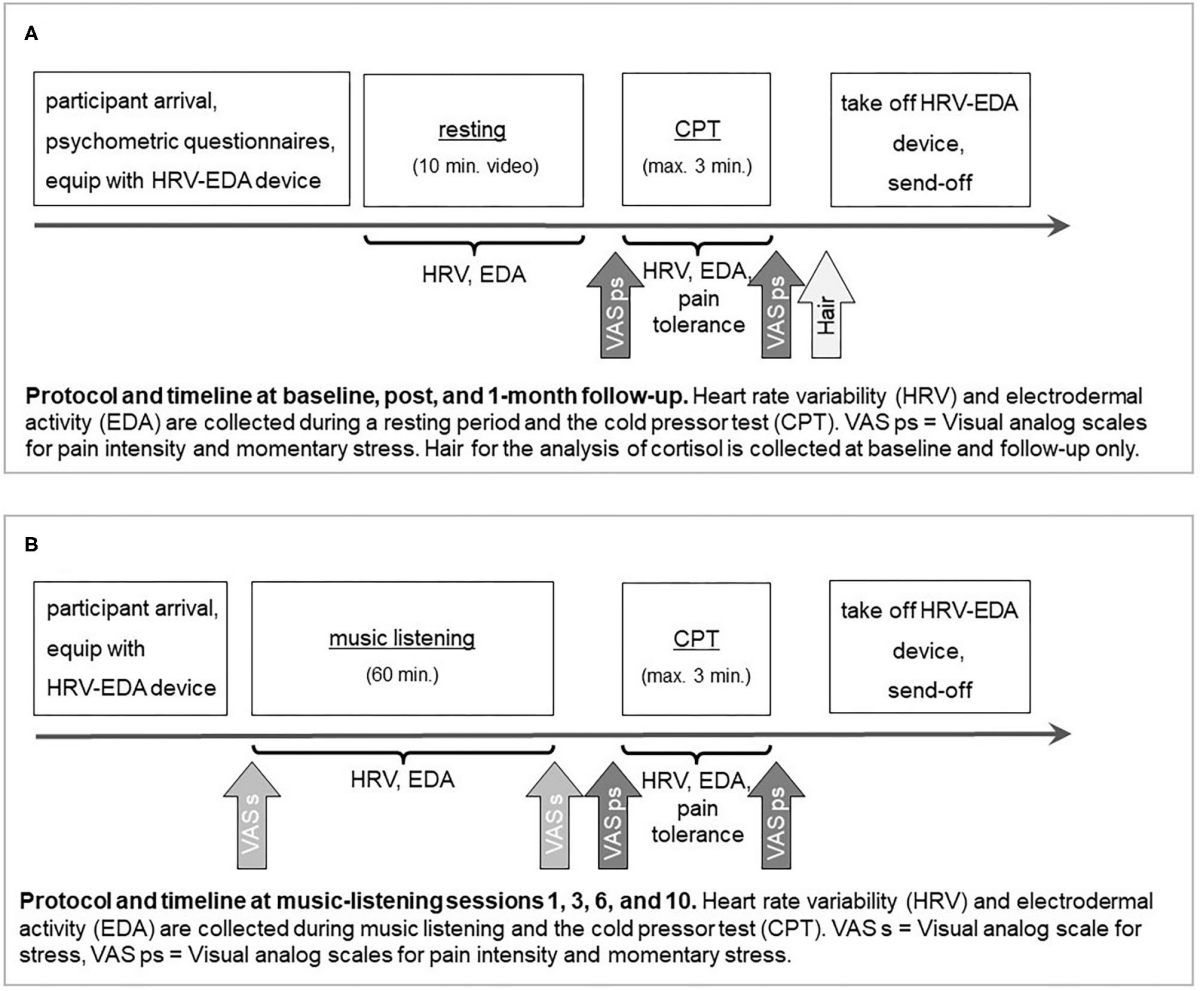
Protocol and timeline for study appointments: **(A)** baseline, post, and follow-up, **(B)** music-listening sessions 1, 3, 6, and 10.

At the beginning of each appointment, participants will be asked to indicate whether any of the behaviors mentioned in Box 1 (drinking alcoholic or energizing beverages, smoking, etc.) apply. Additionally, the examiner will document whether participants state being in a currently stressful phase (i.e., exams), had poor sleep quality or short sleep duration during the previous night, or have any current illness. Affirmation of intake of analgesic medication at baseline, music-listening sessions 1, 3, 6, 10, post-, or follow-up assessments will lead to rescheduling of the respective session since acute pain will be induced at these appointments. The detailed protocol and timeline for baseline, post, and follow-up appointments is displayed in Figure 2A, the protocol and timeline for music-listening sessions 1, 3, 6, and 10 is depicted in Figure 2B. The examiner will be blind regarding frequency modulation and will be in charge of instructing the participants during the course of the study. An independent second scientific staff member, unblinded with regard to intervention conditions, will be in charge of adjusting the music delivery systems (described below) according to the respective music-listening intervention. This person will not interact with the participants face-to-face at any time.

### Study Population

Eligibility criteria are age between 18 and 35 years, body mass index between 18.5 and 30 kg/m^2^, fluency in speaking and reading German, and ability to attend our laboratory for 10 appointments within 3 consecutive weeks. Participants of both genders will be included (15 women and 15 men per group).

The following exclusion criteria are specified to ensure an accurate delivery of the music-listening interventions:

- Perfect pitch
- Music-related studies (i.e., university level education) or profession
- Impairment of hearing capability (e.g., chronic/acute tinnitus, hearing loss).

Additionally, since our primary and secondary outcome measures are susceptible to a range of lifestyle factors and health conditions (98, 99), and in order to reduce any potential risk related to participation in the study, the following further exclusion criteria will be applied based on participant self-report:

- Cardiovascular disease
- Artery occlusive disease
- Hyper-/Hypotension
- Diabetes
- Extreme visual impairment
- Chronic pain condition
- Raynaud syndrome
- Irregular menstrual cycle
- Pregnancy or breastfeeding
- Inability to refrain from smoking for more than 2.5 h
- Regular and problematic alcohol consumption
- Regular intake of pain medication and/or psychotropic drugs
- Mental disorders: current major depression/anxiety disorder; current eating disorder or eating disorder within the past 5 years, current substance abuse or substance abuse within the past 2 years; current or previous psychosis, schizophrenia, or bipolar disorder
- Premenstrual syndrome
- Inability to self-identify as a man or woman.

### Recruitment, Screening, and Consent

Participant recruitment will be conducted by advertising on public notice boards, internet classified ads, social media sites and via announcements in university classes. The study will be presented to the public as “Music for stress management: A music-based intervention study,” and a study email address will be given for contact purposes. Interested potential participants will be asked to send an email including their telephone number in order to establish a first contact.

A two-step screening approach will ensure that only healthy participants are included. First, prospective participants will complete a telephone-based screening interview to check for inclusion and exclusion criteria with regard to medical conditions and lifestyle factors. Thereafter, the detailed study information and an online link to confirm or decline study participation will be sent to the positively screened participants. In the case of confirmation, participants will be emailed a subsequent link leading to an online survey including a battery of questionnaires with further in-depth inclusion and exclusion criteria. If participants screen positive for depression, pre-menstrual syndrome or any psychiatric disorders apply [either based on self-report within the telephone screening or based on the online questionnaires, e.g., Patient Health Questionnaire (100), Beck Depression Inventory-II (101), Premenstrual Syndrome Questionnaire (102)], they will be excluded from the study. If no exclusion criteria apply, participants will be randomly allocated to one of the three music-listening interventions, and the baseline appointment will be scheduled. The informed consent obtained online will be corroborated by a personal signature at baseline. Participants will be compensated for completion of the study with 80 €. In the case of pre-mature termination of the study, participants will be compensated proportionally.

### Randomization

We will use a block randomization stratified by gender using the blockrand package (103) and the statistical software R, version 3.4.2 (104). Block lengths will vary randomly between 3, 6, and 9. The randomization procedure will be performed by an independent researcher unrelated to the study. The resulting 90 computer-generated notes stating participant gender and experimental condition will be placed into separately sealed envelopes and stored in a locked cabinet. Upon inclusion of a new participant, the project coordinator will draw an envelope containing the note with the respective condition to which the included participant will be assigned. This information will be shared with the scientific co-worker who will be in charge of setting the music-delivery systems (see below) during the course of the intervention period, but not with the experimenter.

### Blinding

To ensure that all participants across all conditions have similar beliefs in terms of treatment expectancy, participation in the study (as stated in the study information) implies random assignment to either frequency-modulated or unmodulated music, irrespective of selection of music (researcher- vs. self-selection). The modulation of frequencies in our intervention is not or only barely audible as confirmed in a pilot study conducted in our own lab. Therefore, participants will be unable to detect whether the music they listen to is frequency-modulated (or not), and we will check participants' assumptions concerning their assigned condition at the end of the study (as described below).

With regard to frequency modulation in the researcher-selected conditions, the participants and the examiner (who will be in charge of instructing and interacting with the participants throughout the study sessions) will be fully blinded. Similar to participants in the researcher-selected music conditions, participants in the self-selected music condition will expect to hear either frequency-modulated or unmodulated music. Due to technical constraints, however, frequency modulation will not be applied in this study arm and participants will be exclusively listening to unmodulated music. In this case, the examiner interacting with the participants will be aware of the fact that no frequency modulation will be applied. Consequently, apart from the examiner's and participants' awareness of researcher-selected vs. self-selected music, the study features a double-blind design (examiner and participant) with regard to the frequency modulation within the researcher-selected music arms and a single-blind design (participant only) with regard to the self-selected music arm. The scientific co-worker who will be in charge of the music-delivery systems and administering the correct music intervention to the participating subjects will be fully aware of the respective conditions. Moreover, the study coordinators will also be unblinded since they will be in charge of assigning participants to examiners and scientific co-workers. Neither scientific co-workers nor study coordinators will interact directly with the participants.

### Debriefing

At the end of the post-assessment, participants will be asked to indicate which condition they believe they were allocated to (i.e., frequency-modulated or unmodulated music) in order to check if blinding has been successful. After termination of the study (i.e., after the completion of the follow-up assessment), participants will be debriefed with regard to their intervention allocation. They will be further debriefed with regard to the study goals and the fact that no frequency modulation occurred in the self-selected music-listening condition. In the case of premature study termination, participants will be debriefed accordingly.

### Music-Listening Interventions

Each of the three music-listening interventions consists of 10 sessions of music listening within 3 consecutive weeks. There is no pre-defined minimum number of sessions per week in order to ensure integration of the intervention into participants' schedules. We chose a highly concentrated number of intervention sessions since we assume a dose-dependent effect on measures of pain perception and markers of stress (38, 105). Each music-listening session will encompass 60 min of music listening and 10–20 min for additional data assessments before and after music listening. Participants will receive the intervention individually. During music listening, each participant will be in a reclined position on a lounge chair and will listen to the allocated music via headphones.

#### Frequency-Modulated Researcher-Selected Music

Frequency modulation and music pieces used in this arm are comparable to the procedures of applications of AVWF-based music interventions in previous clinical studies (74, 75). Six different mixes of music pieces are chosen that cover a wide range of genres such as classic, instrumental, pop, rock, and world music (see Box 2 for details). Three of these mixes are compositions of known artists of which two also contain vocals. The remaining three of the music mixes are instrumental music pieces that were directly composed, recorded, and provided by the developer of the AVWF method. Four of the six mixes will be presented repeatedly within the 10 music-listening sessions. Each mix has a length of 60 min. According to the AVWF method, the applied music is modulated within the audible frequency range of 50–4,000 Hz via a software system. This involves filtering the harmonic overtones of low frequencies of the music pieces presented in music-listening sessions 1–7. In sessions 8–10, modulation will be additionally applied to frequencies in the high spectrum. The modulated music will be transferred onto a music delivery system (described below) which is equipped with hardware components and additional modulating features, increasing the magnitude of modulation from session to session. This treatment is based on the assumption that listening to music modulated in the low- and high-frequency spectrum improves stress regulation and benefits the ANS via indirect stimulation of afferent and efferent nerves within the auditory passage (106).

Box 2 **Overview of the music used in each music-listening session in the researcher-selected music conditions.**

**Table:** 

**ML session**	**Title of Album(s)**	**Artist(s)**
1	Well-balanced	Oliver Shanti
2	AVWF–Classics I	AVWF®
3	QE2; Earth Moving	Mike Oldfield
4	Violine Volume I	AVWF®
5	The Beatles; The Beatles	Munich Symphonic Sound Orchestra; Classic Dream Orchestra
6	See session 1	
7	See session 4	
8	See session 5	
9	See session 3	
10	Guitar I	AVWF®
Participants will listen to a mix of music pieces of the specified album and artist for 60 min in each session. Frequency modulation increases with session number in the frequency-modulated researcher-selected music-listening (ML) condition.		

For persons with average hearing ability, the frequency modulation is typically not detectable according to the AVWF developer. We conducted a pilot study with a convenience sample of 10 healthy subjects (seven women, three men, all psychology students) in order to test whether participants would be able to guess correctly if they listened to frequency-modulated or unmodulated music. We randomly assigned participants to one of the two conditions (5 per group) and chose a composition of classic instrumental music that was self-composed by the AVWF developer for both conditions. Moreover, for the frequency-modulated music condition, we decided to apply a modulation stage that would be usually played during the sixth session (i.e., advanced modulation of frequencies compared to the first sessions). Participants listened to the respective music for a duration of 20 min in sitting position via headphones. Afterwards, they filled out a paper-pencil questionnaire asking them (a) to indicate whether the music they listened to was presumably frequency-modulated or unmodulated and (b) to estimate how confident they feel in their answer on a VAS ranging from 0 to 100%. One participant did not provide answers, leaving data from nine participants for evaluation. Three participants (33.3%) allocated to the frequency-modulated music made the correct guess with an average confidence level of 50.3%. It is notable that one of these three participants had visual impairments and reported extremely good hearing abilities. Moreover, three participants (33.3%) who were allocated to unmodulated music, guessed wrongly and reported having been listening to frequency-modulated music with an average confidence level of 43.0%. Furthermore, one participant (11.1%) reported correctly having listened to unmodulated music with a confidence level of 15.0% and two participants (22.2%) thought they listened to unmodulated music although they had been allocated to the frequency-modulated music, with an average confidence level of 42.5%. In summary, considering the 50% chance of guessing correctly and that those who did guess correctly were not overall confident in their estimation, we concluded from these results that there was no systematic identification of frequency-modulated music, confirming the notion that frequency modulation is typically undetectable.

#### Unmodulated Researcher-Selected Music

The same music pieces in identical sequence as in the frequency-modulated researcher-selected music condition (see Box 2) will be presented to participants. However, music pieces will not be frequency-modulated and will be presented via a music delivery system without modulating features.

#### Unmodulated Self-Selected Music

Participants will receive an email before their baseline appointment instructing them to compile 120 min of their favorite music, irrespective of genre or other musical characteristics (e.g., tempo, instrumental, vocal). Participants will be assured that their personal selection will not be judged in any way and that they should select songs or musical excerpts that they will be able to enjoy listening to for 60 min. Participants will also be advised to bring their compilation on a portable memory device in mp3 format at their baseline appointment, when the examiner will transfer the music onto the study server and onto the music-delivery system without modulating features for the subsequent 10 music-listening sessions. During the intervention period, the self-selected music will be played in shuffle mode in order to ensure variation across and within sessions. On every occasion, the current playlist will be recorded by the scientific co-worker for our internal records.

### Materials and Equipment

#### Music-Delivery Systems

Music will be presented via two different non-commercially available music-delivery systems [as described in (75)]. One of these will modulate the music via specific hardware components while the other will play music unaltered. Both systems consist of a computer with touch display and an amplifier built into a wooden case and equipped with Windows media player software. All participants will listen to their respective music via over-ear headphones (beyerdynamic, Heilbronn, Germany).

#### Pain Induction

The cold pressor test (CPT) is a safe, reliable, and frequently applied method to induce cold pain in experimental settings (107, 108). The CPT apparatus consists of a plastic bucket filled with crushed ice and water. A metal grid placed into the bucket holds the ice at the bottom of the bucket, and an electrical pump constantly circulates the water to maintain a constant temperature within the bucket. The target temperature is 1°C and is controlled by a thermometer. Participants are asked to immerse their dominant hand into the water up to their wrist without moving their hand or making a fist. Participants are instructed to keep their hand in the water for as long as they can stand it. In this study, participants will be facing a wall, and the examiner will turn away from the participant in order to eliminate confounding due to social desirability. When extracting their hand from the water, the participants will be asked to signal this verbally to the examiner. After a maximum duration of 3 min, participants will be asked to pull their hand out of the water in order to prevent any potential tissue damage. Moreover, participants do not know after which of the music-listening sessions they will be exposed to the CPT in order to avoid anticipation effects.

#### Outcome Measures

##### Course of Assessments

Table 1 reports the assessment schedule for measures of pain perception as well as for biological and subjective markers of stress. See Figure 2A for the protocol and timeline for baseline, post, and follow-up appointments and Figure 2B for the protocol and timeline for music-listening sessions 1, 3, 6, and 10. In accordance with our hypotheses regarding measures of pain perception and markers of stress, primary and secondary outcomes will be measured at baseline and post-intervention. Furthermore, to investigate the intermediate stability of potential benefits of the music-listening interventions, we have included a follow-up assessment 4 weeks after the post-assessment.

**Table 1:** Assessment schedule for measures of pain perception and markers of stress.

**Measures**		**Online survey**	**BL**	**Intervention period**										**Post**	**FU**
				**M1**	**M2**	**M3**	**M4**	**M5**	**M6**	**M7**	**M8**	**M9**	**M10**		
Pain perception	Pain tolerance (seconds)		x	x		x			x				x	x	x
	Perceived pain intensity^a^ (VAS)		x	x		x			x				x	x	x
Subjective stress	Momentary stress^b^ (VAS)		x	x	x	x	x	x	x	x	x	x	x	x	x
	Chronic Stress (SSCS)	x												x	x
	Stress reactivity (PSRS)	x												x	x
Biological stress markers	HRV^c^		x	x		x			x				x	x	x
	EDA^c^		x	x		x			x				x	x	x
	Hair cortisol		x												x

*The intervention period comprises 10 sessions of music listening (for a duration of 60 min each) scheduled within 3 consecutive weeks. Perceived pain will be induced via the cold pressor test (CPT) at baseline (BL), music-listening sessions (M) 1, 3, 6, and 10 (after music listening, respectively), at post, and at follow-up (FU). Music-listening sessions 2, 4, 5, 7, 8, and 9 comprise music listening only and no subsequent pain induction*.

a *Perceived pain intensity will be assessed pre- and post-CPT*.

b *Momentary stress will be assessed pre- and post-music listening and pre- and post-CPT*.

c *HRV and EDA will be derived from resting state (=10 min) at baseline, post, and follow-up; in addition, HRV and EDA will be measured continuously throughout music-listening sessions 1, 3, 6, and 10*.

*EDA, electrodermal activity; HRV, heart rate variability; PSRS, Perceived Stress Reactivity Scale; SSCS, Screening Scale for Chronic Stress; VAS, visual analog scale*.

The intervention period encompasses 10 sessions of music listening, each for a duration of 60 min, within 3 consecutive weeks. Music-listening sessions 1, 3, 6, and 10 will also include assessments of perceived pain in response to cold pain (induced via the CPT after music listening) and continuous measurements of biomarkers of the ANS as well as measurements of momentary subjective stress before and after music listening, as well as before and after the CPT. Furthermore, we will assess a range of tertiary variables during the course of the study (e.g., music-induced emotions, mood). The detailed assessment schedule including all measured variables (primary, secondary, tertiary) is reported in the Supplementary Tables 1, 2.

##### Primary Outcomes

Pain tolerance and perceived pain intensity: Pain tolerance will be operationalized via time in seconds elapsed from immersion until extraction of the hand during the CPT. It will be recorded by the examiner using a stopwatch. Perceived pain intensity will be measured before and after the CPT via paper-and-pencil visual analog scales (VAS). Participants will be provided with a piece of paper containing the sentence “I am in pain” and a 10 cm line ranging from 0 to 100. They will be instructed to mark the line accordingly (0 = no pain, 100 = maximal pain). Additionally, after the CPT, participants will be asked to report how painful they perceived the CPT to be by responding to the sentence “The test was painful” and again marking a VAS corresponding to their experience (0 = not at all painful, 100 = maximally painful). Measures of pain perception will be assessed at baseline, post, and follow-up as well as after music listening in sessions 1, 3, 6 and 10 (see Table 1 and Figure 2).

##### Secondary Outcomes

Since stress is a multidimensional phenomenon, we consider biological (autonomic, endocrine) as well as subjective indicators that operationalize different aspects and time-varying characteristics of stress as our secondary outcomes (109).

##### Autonomic and Endocrine Stress Markers

###### Heart Rate Variability

We will record an electrocardiogram (ECG) at a sampling rate of 256 Hz for the analysis of heart rate variability as an indicator of ANS activity using Equivital EQ02 Life Monitors and Equivital Life Shirts (Hidalgo Limited, Cambridge, UK). Time domain (e.g., square root of the mean squared differences between successive RR intervals, RMSSD; percentage of successive RR intervals that differ by more than 50 ms, pNN50) as well as frequency-domain (e.g., high-frequency band, HF; low- to high-frequency ratio, LF/HF) indices (110) will be calculated. At baseline, post, and follow-up, ECG recordings will take place at rest for 10 min while watching a video featuring landscapes (111) with the sound turned off. At the same appointments, ECG will be additionally recorded continuously (i.e., including pain induction). In music-listening sessions 1, 3, 6, and 10, ECG will be recorded constantly throughout the whole session (i.e., including music listening and pain induction).

###### Electrodermal Activity

Electrodermal activity (EDA) will be recorded at a sampling rate of 16 Hz using Equivital EQ02 Life Monitors and corresponding EQ-ACC-034 EDA sensors (Hidalgo Limited, Cambridge, UK). EDA will be derived from the intermediate phalanx of the middle and index finger of the non-dominant hand using pre-gelled Biopac EL507 electrodes with Ag/AgCl contacts (Biopac Systems Inc., Goleta, CA, USA). Recording will take place analogously to ECG recordings.

###### Hair Cortisol

Cortisol is the main effector hormone of the hypothalamic-pituitary adrenal axis, an important stress-sensitive system besides the ANS. The secretion of cortisol is increased upon exposure to environmental stressful situations and accumulates in hair, reflecting a measure of chronic stress exposure (112). Hair samples for the subsequent analysis of hair cortisol will be taken at baseline and follow-up assessment. Three strands of hair will be taken scalp-near from the posterior vertex region. The most scalp-near 1 cm of hair will be analyzed as this represents cortisol secretion in approximately the last month and thus gives insight into each individual's cumulative stress exposure over the month before sampling. In the context of this study, we will thus be able to assess chronic biological stress in the 4 weeks before baseline and in the 4 weeks before follow-up (reflecting the timeframe between the post-measurement and follow-up assessment).

##### Subjective Stress Measures

###### Subjective Momentary Stress

We will measure momentary subjective stress via VAS (paper-and-pencil) before and after music listening in all 10 music-listening sessions and before and after conducting the CPT (i.e., at baseline, music-listening sessions 1, 3, 6, 10, post, and follow-up). On each occasion, participants will be asked to respond to the sentence “I am feeling stressed” using a VAS ranging from 0 (not stressed at all) to 100 (maximally stressed).

###### Chronic Stress

We will use the Screening Scale for Chronic Stress (SSCS) comprising 12 items of the Trier Inventory of Chronic Stress (TICS) (113) at baseline, post, and follow-up assessment to measure subjective chronic stress. We have adjusted the reference period in the instructions of the SSCS from 4 weeks to 1 week so that baseline and post-assessment will not overlap. The TICS encompasses 57 items on six subscales (work overload, worries, social stress, lack of social recognition, work discontent, intrusive memories) and will be used at baseline, too.

###### Stress Reactivity

Stress reactivity will be measured by the German version of the Perceived Stress Reactivity Scale (PSRS) (114), administered at baseline, post, and follow-up assessment. The PSRS consists of five subscales (Prolonged Reactivity, Reactivity to Work Overload, Reactivity to Social Conflicts, Reactivity to Failure, Reactivity to Social Evaluation) which can be combined into one overall scale.

##### Tertiary Variables

Tertiary variables will be assessed throughout the study. These may serve as outcome, control, moderator and/or mediator variables in exploratory analyses related to the study. The assessment schedule for tertiary variables is displayed in the Supplementary Tables 1, 2.

In each music-listening session, momentary mood [short scale of the Multidimensional Mood Questionnaire, MDMQ-short (115)] will be assessed directly before and after music listening. Additionally, based on previous literature investigating the impact of music on measures of pain perception and stress, we developed a questionnaire assessing music-related perceptions concerning the respective music that was listened to. The questionnaire will be provided after music listening in each session. It includes questions on music-induced emotions, perceived valence and arousal of the music, music-induced memories, and is also thought to control for mind-wandering or sleeping during the music-listening sessions (see questionnaire 1 in online Supplementary Material). Furthermore, musical engagement between sessions will be assessed (see questionnaire 2 in online Supplementary Material) as a potential control variable.

At baseline, post, and follow-up assessments, the following additional variables will be assessed via questionnaires: emotion regulation strategies [Emotion Regulation Questionnaire, ERQ (116)], fatigue [Multidimensional Fatigue Inventory, MFI-20 (117)], sleep quality [Pittsburgh Sleep Quality Index, PSQI (118)], and depressive symptoms [Beck Depression Inventory-II, BDI-II (101)]. Moreover, at baseline only, cognitive style of music listening (Music-Empathizing-Music-Systemizing (ME-MS) Inventory), music preferences [revised version of the Music Preference Questionnaire, MPQ (119)], personality traits [openness to experience, conscientiousness, extraversion, agreeableness, neuroticism, Big Five Inventory-10 (120)], perceived social support [subscale of the Berlin Social Support Scales, BSSS (121)], and menstrual cycle phase (self-report) will be assessed.

For the purpose of monitoring the participants during the intervention period, they will receive paper-and-pencil questionnaires with open-ended questions at the end of music-listening sessions 1, 3, 6, and 10. This will enable them to report whether they felt any disturbances associated with the music-listening session and whether they had any specific thoughts during the CPT in order to control for cognitive pain-coping strategies.

At the end of the post-assessment, participants will receive a paper-and-pencil post-monitoring questionnaire, asking them to indicate which condition (i.e., frequency-modulated or unmodulated music) they believe they were assigned to and how confident they are in this belief. Moreover, participants will be asked whether they perceived increased self-awareness due to study participation, whether any positive or negative changes in well-being, mood, or general health occurred during the course of the intervention, and to state how compatible study participation was with their individual schedules. At the follow-up assessment, final post-monitoring and control questions regarding musical engagement, previous familiarity with and use of music-listening interventions and study conformity will be assessed.

### Sample Size Determination

Previous investigations employing a comparable design are lacking and we consider the current study a pilot trial in order to yield sufficient precision for a sample size calculation in a subsequent full trial. Therefore, following the recommendations mentioned in (122, 123) and (124), we decided to test 30 participants in each group. A power analysis using G^*^Power 3 (125) suggested that this sample size will be sufficient to achieve a medium effect (*f* = 0.25) in a repeated-measures analysis of variance (α = 0.05, power (1-β) = 0.80) with condition as the between-subject factor (frequency-modulated researcher-selected vs. unmodulated researcher-selected vs. self-selected) and time (baseline vs. post) as the within-subject factor. In the case of dropouts, we will continue recruitment and repeat the randomization procedure until at least a number of 30 participants is collected in each group. Thus, the final number of participants in each group might slightly differ.

### Statistical Analysis

Repeated-measures analyses of variance will be conducted to test our main hypotheses 1 and 2. Mediation hypotheses will be tested with continuous time modeling procedures. *P*-values of ≤ 0.05 will be considered statistically significant. Analyses for comparisons between the researcher-selected frequency-modulated and self-selected unmodulated music conditions will be conducted in a two-sided manner. Any further exploratory analyses will be specified in future publications. We will perform both per-protocol and intention-to-treat analyses. Missing data will be imputed in accordance with (126).

### Ethical Considerations

The study has been approved by the Local Ethics Committee of the Department of Psychology of the University of Marburg (2016–27k) and the Local Ethics Committee of the University of Vienna (00331). It is preregistered on ClinicalTrials.gov (Identifier: NCT02991014). The study has begun in Marburg (recruitment and testing from December 2016 until December 2017) and will be continued in Vienna. Potential participants will be informed about the procedure and general aims of the study. Participants will be told that they will be randomly assigned to a researcher-selected or self-selected music-listening condition; they will be told that their respective music might be frequency-modulated. In fact, only researcher-selected music will be frequency-modulated. This deception is necessary to avoid any confounding effects due to expectancy bias. Written informed consent will be obtained from all participants. Participants can withdraw from the study at any time. All participants will be debriefed upon completion of or withdrawal from the study.

Conducting the CPT will be painful. Nevertheless, in order to study effects of music-based interventions on pain, it is essential to evoke pain. Participants can withdraw their hand at any time. Maximum immersion time will be limited to 3 min to ensure the safety of the procedure. To further rule out any risk, persons suffering from conditions like cardiovascular diseases, Raynaud syndrome or high or extremely low blood pressure will be excluded from participation.

### Dissemination

Results of this research study will be presented at national and international conferences and published in a peer-reviewed journal. In accordance with the recommendations of the German Psychological Association (DGPs) (127), primary data of this study will be made available in an electronic online repository.

### Data Monitoring and Management

A data monitoring committee has not been established since this study is considered to be of minimal risk. Questionnaires that are administered electronically will be saved in a password-protected online database. Paper-and-pencil questionnaires will be stored in a locked cabinet. They will be entered into and saved in electronic files on a regular basis. Access to study data will be limited to research staff who require direct access.

### Confidentiality

After inclusion of a new participant (i.e., after online confirmation of study participation and successful online screening), the participant will be allocated a unique study code (sequential number), which will be used for all further study documentation to ensure confidentiality. All data analysis will be performed via study code only. The master files that connect the unique participant codes with sensitive person-related information will be stored separately in a locked cabinet with limited access.

## Discussion

Music-listening interventions are an effective adjuvant for the management of pain and stress. There is still uncertainty concerning the role of particular treatment characteristics for the effects of music-listening interventions on measures of pain perception and markers of stress. Some authors argue that certain frequencies in music might be especially useful for alleviating perceived pain and stress, and suggest that musical stimuli should be modulated accordingly. Moreover, some researchers emphasize the importance of high standardization in the design of music-listening interventions and thus argue in favor of music selection by researchers, while others advocate self-selection of musical stimuli by participants to achieve beneficial effects. Furthermore, very few studies have investigated both measures of pain perception and markers of stress together, making it difficult to unravel the potential role of stress in pain perception.

This study aims to determine potential influences of frequency modulation as well as of self- vs. researcher-driven selection of music stimuli in terms of the efficacy in reducing perceived pain and markers of stress. Moreover, while there is comprehensive evidence of direct effects of music-based interventions on pain perception, we seek to investigate whether these effects might be mediated by changes in biomarkers of the ANS and subjective stress and whether this can result in long-term benefits. To address these open questions, we designed a randomized controlled, laboratory-based, double-blind pilot trial comparing frequency-modulated researcher-selected music with unmodulated researcher-selected music. In addition, we included a third condition, in which participants will listen to their self-selected unmodulated music.

Certain limitations of our study warrant consideration. Due to technical constraints, we will not have the opportunity to conduct frequency modulation on music, which is self-selected by participants. Thus, our design does not allow us to test a possible interaction between frequency modulation (modulated or unmodulated) and selection (self-selected or researcher-selected). In addition, the procedures for participants randomized to the self-selected music condition are not entirely comparable to the procedures for participants in the researcher-selected music conditions: participants in the self-selected music condition will spend time and effort in selecting their favorite pieces of music whereas this does not apply to participants in the researcher-selected music condition. In case of significant differences between these two conditions, we cannot completely rule out that the effects depend, in part, on the act of listening to music that one has chosen in an effortful process or other confounding aspects instead of the music *per se*. Examining these mechanisms lies, however, beyond the scope of the current study and has been discussed elsewhere with suggestions on how to disentangle the confounding variables (e.g., increased control over choice, familiarity) in studies investigating self- and researcher-selected music (10). Furthermore, due to the repeated exposure to the CPT, the possibility of desensitization effects needs to be considered. There are not many studies that investigated intervention effects on cold pressor pain including a repeated exposure to the CPT. However, those that did, found that there were no desensitization effects with respect to pain tolerance (128). Furthermore, considering biomarkers of the ANS, Minkley et al. (129) found no desensitization effects on blood pressure during repeated exposure to the (socially evaluated) CPT, however, desensitization of heart rate was documented. Other studies suggest that ANS activity remains unaffected by repeated exposure to the cold pressor test if the recovery period is long enough (130), which applies to the present study. Importantly, if desensitization effects should occur in the present study, we would assume that these affect participants across conditions due to the randomization and would thus apply to all three conditions. Therefore, systematic differences in pain perception between the conditions should stay unaffected. Moreover, we are only able to investigate the effect of one specific method of frequency modulation. However, we chose to investigate this particular approach, since the AVWF method has been applied in clinical research with patients suffering from stress-associated disorders with first positive results (74, 75). Consequently, it seems a reasonable starting point to test specifically the AVWF method and to investigate its potential to reduce perceived pain and markers of stress in a pilot-RCT. Another limitation concerns the lack of a non-musical control condition (e.g., listening to an audio book or another type of non-musical auditory stimulation). However, adding such a group would be of limited use because this would merely allow us to test whether music *per se* has an effect compared to non-musical auditory stimuli. The effectiveness of music *per se* in reducing pain (14, 17) and stress (39, 41) has already been shown in various studies. Thus, given the costs of including an additional control group, we did not deem such a group necessary in order to answer our study questions within the current pilot-RCT. Furthermore, considering the 4 weeks between the post- and follow-up assessments, it might be argued that this is a rather short timeframe in order to investigate the stability of potential benefits of the music-listening interventions. However, in the context of our study, stability needs to be considered in the context of the experimental nature of the study, such that it might provide initial evidence on how long potential effects could last in healthy participants. If our results reveal preliminary evidence for effects enduring for 4 weeks after termination of the interventions, this would clearly underscore the importance of further examinations of such effects in a subsequent study, which may include chronic pain patients and a longer duration until follow-up assessment. Finally, we will only recruit healthy young adults in a laboratory setting using experimentally induced pain, which obviously differs from real-life pain conditions in several ways, such as the individual's possibility to stop the pain-inducing procedure at any time (19). Thus, the generalizability of findings will be limited. Nevertheless, in order to investigate biopsychological mechanisms, we consider it important to examine potential effects in a well-controlled, highly standardized design first, before conducting studies with clinical populations in more naturalistic settings.

Apart from the above-mentioned limitations, there are several strengths of our study. To the best of our knowledge, this is the first study to test effects of frequency-modulated music vs. researcher-selected music on experimentally induced acute pain using a longitudinal, randomized controlled and double-blind design. By including a third condition, i.e., self-selected unmodulated music we will be able to compare the differential effects of the three music-listening interventions and to fill research gaps that have been documented in the literature (9, 10, 14). Moreover, by including measures of biological and subjective stress, the current study will address secondary variables that have been proposed to mediate the effects of music on pain perception (37, 38). Unlike most previous studies, we will not investigate effects on measures of pain perception during concurrent music listening but will instead test whether even after cessation of listening to music an effect on pain perception can still be achieved. This will enable us to investigate the potential stability of the pain-reducing effects of music listening—at least within an intermediate time-frame, an aspect that is currently highly under-researched (14). Moreover, we will measure a great variety of tertiary variables such as sleep quality, fatigue, mood, and music-related perceptions, allowing us insights into the effects of music on many domains of health.

Consequently, this study is the first important step toward a deeper understanding of the efficacy, the role of treatment characteristics (i.e., frequency modulation, control over selection of music) and biopsychological mechanisms underlying the phenomenon of music-induced analgesia. The results from the current pilot-RCT will provide important information on the differential effects and effect-sizes on perceived pain as well as the potential biopsychological mechanisms underlying the effects of the three employed music-listening interventions on perceived pain. These findings are pivotal for the sound design of future large-scale randomized controlled trials focusing on the effects of particular music-listening interventions for the reduction of pain. In addition, although preliminary, results from this study will be highly informative for the implementation and improvements of music-listening interventions offered to acute and chronic pain patients.

## Ethics Statement

The study has been reviewed and approved by the Local Ethics Commitee, Department of Psychology, University of Marburg, Marburg, Germany (2016–27k), and the Local Ethics Commitee, University of Vienna, Vienna, Austria (00331). All participants will provide their written informed consent to participate in this study.

## Author Contributions

AF, MK, BD, DO, and UN designed the study. AF, MK, and RM wrote the first draft of the manuscript. All authors reviewed and edited the manuscript and approved its final version.

## Conflict of Interest

The authors declare that the research was conducted in the absence of any commercial or financial relationships that could be construed as a potential conflict of interest.

## Footnotes

^1^A corresponding English translation is “Audiovisual Perception Enhancement”.
