# Supplementary material for: Efficacy, Treatment Characteristics, and Biopsychological Mechanisms of Music-Listening Interventions in Reducing Pain (MINTREP): Study Protocol of a Three-Armed Pilot Randomized Controlled Trial
Source: Front Psychiatry. 2020 Nov 4;11:518316. doi: 10.3389/fpsyt.2020.518316 (PMC7672017; doi:10.3389/fpsyt.2020.518316)
Supplement: Supplementary file 1 [file Data_Sheet_1.docx]

Supplementary Material

**“Efficacy, Treatment Characteristics, and Biopsychological Mechanisms of Music-Listening Interventions in Reducing Pain (MINTREP): Study Protocol of a Three-Armed Pilot Randomized Controlled Trial”**

Table of Contents

[TABLE 1 Detailed assessment schedule for all study appointments. 2](#_Toc51438101)

[TABLE 2 Detailed assessment schedule for music-listening sessions 1, 3, 6, and 10 4](#_Toc51438102)

[QUESTIONNAIRE 1 Music-related perceptions 5](#_Toc51438103)

[QUESTIONNAIRE 2 Musical engagement between music-listening sessions 8](#_Toc51438104)

[References 9](#_Toc51438105)

| TABLE 1 Detailed assessment schedule for all study appointments. | | | | | | | | | | | | | | | | |
| --- | --- | --- | --- | --- | --- | --- | --- | --- | --- | --- | --- | --- | --- | --- | --- | --- |
| Measures |  | | **Online**  **survey** | **BL** | **Intervention period** | | | | | | | | | | **Post** | **FU** |
|  |  |  |  |  | M1 | M2 | M3 | M4 | M5 | M6 | M7 | M8 | M9 | M10 |  |  |
| *Primary outcomes* | | |  |  |  |  |  |  |  |  |  |  |  |  |  |  |
| Pain | | Pain tolerance (seconds) |  | x | x |  | x |  |  | x |  |  |  | x | x | x |
|  | | Perceived pain intensity^1^ (VAS) |  | x | x |  | x |  |  | x |  |  |  | x | x | x |
| *Secondary outcomes* | | |  |  |  |  |  |  |  |  |  |  |  |  |  |  |
| Subjective stress | | Momentary stress**^2^** (VAS) |  | x | x | x | x | x | x | x | x | x | x | x | x | x |
|  | | Chronic Stress  (SSCS) | x |  |  |  |  |  |  |  |  |  |  |  | x | x |
|  | | Stress reactivity  (PSRS) | x |  |  |  |  |  |  |  |  |  |  |  | x | x |
| Biological stress markers | | HRV indices^3^ |  | x | x |  | x |  |  | x |  |  |  | x | x | x |
|  | | EDA^3^ |  | x | x |  | x |  |  | x |  |  |  | x | x | x |
|  | | Hair cortisol |  | x |  |  |  |  |  |  |  |  |  |  |  | x |
| *Tertiary variables* | | |  |  |  |  |  |  |  |  |  |  |  |  |  |  |
| Momentary mood^4^ | | MDMQ-short |  | x | x | x | x | x | x | x | x | x | x | x | x | x |
| Music-related perceptions^5^ | | self-developed |  |  | x | x | x | x | x | x | x | x | x | x |  |  |
| Musical engagement between sessions^5^ | | self-developed |  |  | x | x | x | x | x | x | x | x | x | x | x | x |
| Music preferences | | MPQ-R | x |  |  |  |  |  |  |  |  |  |  |  |  |  |
| Table 1 (continued) | | | | | | | | | | | | | | | | |
| Measures | |  | **Online survey** | **BL** | **Intervention period** | | | | | | | | | | **P** | **FU** |
|  |  |  |  |  | M1 | M2 | M3 | M4 | M5 | M6 | M7 | M8 | M9 | M10 |  |  |
| Cognitive style of music listening | | ME-MS | x |  |  |  |  |  |  |  |  |  |  |  |  |  |
| Mood regulation via music | | B-MMR | x |  |  |  |  |  |  |  |  |  |  |  | x | x |
| Emotion regulation | | ERQ | x |  |  |  |  |  |  |  |  |  |  |  | x | x |
| Depression | | BDI-II | x | x |  |  |  |  |  |  |  |  |  |  | x | x |
| Fatigue | | MFI-20 | x |  |  |  |  |  |  |  |  |  |  |  | x | x |
| Sleep Quality | | PSQI | x |  |  |  |  |  |  |  |  |  |  |  | x | x |
| Chronic Stress | | TICS | x |  |  |  |  |  |  |  |  |  |  |  |  |  |
| Personality | | BFI-10 | x |  |  |  |  |  |  |  |  |  |  |  |  |  |
| Perceived social support | | BSSS  (subscale) | x |  |  |  |  |  |  |  |  |  |  |  |  |  |
| Mental health | | PHQ-D | x |  |  |  |  |  |  |  |  |  |  |  |  |  |
| Premenstrual syndrome | | PMS questionnaire | x |  |  |  |  |  |  |  |  |  |  |  |  |  |
| Menstrual cycle | |  | x |  |  |  |  |  |  |  |  |  |  |  |  |  |
| Hair characteristics | |  |  | x |  |  |  |  |  |  |  |  |  |  |  | x |
| ^1^ Perceived pain intensity will be assessed pre- and post-pain induction via cold pressor test (CPT);  ^2^ Momentary stress will be assessed pre- and post-music listening and pre- and post-CPT;  ^3^ HRV and EDA: resting state (=10 minutes) at baseline, post, and follow-up; continuous measurement throughout music-listening sessions 1, 3, 6, and 10 (during music listening and pain induction);  ^4^ Momentary mood will be assessed pre- and post-music listening;  ^5^ Self-developed questionnaire (see below).  BL, baseline assessment; FU, follow-up assessment; M1 to M10, music-listening sessions 1 to 10; VAS, visual analog scale; SSCS, Screening Scale for Chronic Stress (1); TICS, Trier Inventory of Chronic Stress (1); PSRS, Perceived Stress Reactivity Scale (2); HRV, heart rate variability; EDA, electrodermal activity; MDMQ-short, short-scale of the Multidimensional Mood Questionnaire (3); MPQ-R, Music Preference Questionnaire, revised version (4); ME-MS, Music-Empathizing-Music-Systemizing Inventory (5); B-MMR, Brief Music in Mood Regulation Scale (6); ERQ, Emotion Regulation Questionnaire (7); MFI-20, Multidimensional Fatigue Inventory (8); PSQI, Pittsburgh Sleep Quality Index (9); BFI-10, Big-Five-Inventory-10 (10); BSSS, Berlin Social Support Scale (11); BDI-II, Beck Depression Inventory II (12); PHQ-D, German version of the Patient Health Questionnaire (13); PMS questionnaire, Premenstrual Syndrome Questionnaire (14). | | | | | | | | | | | | | | | | |

| **TABLE** 2 Detailed assessment schedule for music-listening sessions 1, 3, 6, and 10 | | | | | | |  |
| --- | --- | --- | --- | --- | --- | --- | --- |
| **Measures** | **before ML** | **during ML** | **after ML** | **before CPT** | **during CPT** | **after CPT** | |
| HRV^1^ | x | x | x | x | x | x | |
| EDA^1^ | x | x | x | x | x | x | |
| Pain tolerance (seconds) |  |  |  |  | x |  | |
| Perceived pain intensity (VAS) |  |  |  | x |  | x | |
| Momentary stress  (VAS) | x |  | x | x |  | x | |
| Momentary mood  (MDMQ-short) | x |  | x |  |  |  | |
| Music-related perceptions^2^ |  |  | x |  |  |  | |

Participants will listen to music for 60 minutes in all sessions, respectively. ML sessions 2, 4, 5, 7 – 9 include only music listening and no pain induction and comprise assessments of subjective momentary stress, mood, and music-related perceptions only before/after music listening;

^1^ Continuous measurement throughout the complete session;

^2^ Self-developed questionnaire (see below);

EDA, electrodermal activity; HRV, heart rate variability; MDMQ-short, short-scale of the Multidimensional Mood Questionnaire (3); ML, music listening; VAS, visual analog scale.

# **QUESTIONNAIRE 1** Music-related perceptions

The following questions refer to the music you have listened to during today’s appointment. There are no right or wrong answers. Please answer the following questions according to your personal appraisals.

|  | **not at all** | **1** | **2** | **3** | **4** | **5** | **very much** |
| --- | --- | --- | --- | --- | --- | --- | --- |
| I liked the music. | not at all | O | O | O | O | O | very much |
| The music was familiar to me. | not at all | O | O | O | O | O | very much |
| I was focusing on the music. | not at all | O | O | O | O | O | very much |
| The music disturbed me. | not at all | O | O | O | O | O | very much |
| The music helped me to relax. | not at all | O | O | O | O | O | very much |
| The music helped me to take my mind off things. | not at all | O | O | O | O | O | very much |
| The music activated me. | not at all | O | O | O | O | O | very much |

For the following questions, please draw a short vertical line at the point on the line that best describes your *personal evaluation*.

The music was …

happy sad

relaxing energizing

Do you associate specific memories with the music you listened to?

No o

Yes o

**QUESTIONNAIRE 1** Music-related perceptions (continued)

if so: The memories that were triggered by the music were

positive negative

What did you concentrate on while listening to the music? (please tick one option only)

O Music

O Memories

O Both (music and memories)

O Other

O None of the above

Which emotions did the music elicit in you?

|  | **not at all** | **1** | **2** | **3** | **4** | **5** | **very much** |
| --- | --- | --- | --- | --- | --- | --- | --- |
| Joy | not at all | O | O | O | O | O | very much |
| Sadness | not at all | O | O | O | O | O | very much |
| Relaxation | not at all | O | O | O | O | O | very much |
| Anger | not at all | O | O | O | O | O | very much |
| Fear | not at all | O | O | O | O | O | very much |
| Nostalgia | not at all | O | O | O | O | O | very much |
| Melancholia | not at all | O | O | O | O | O | very much |

What did you pay attention to while listening to the music?

O During the 60 minutes I spent more time thinking about things other than the music I listened to (e.g., memories, plans for the future, daydreaming, etc.).

O During the 60 minutes I mainly focused on the music.

O Other (please describe): ______________________________________________

**QUESTIONNAIRE 1** Music-related perceptions (continued)

Please mark which of the following statements applies best to you:

O I slept for more than 30 minutes while listening to the music.

O I slept while listening to the music, but for less than 30 minutes.

O I did not sleep while listening to the music.

Experience of chills

Chills are physical reactions that are typically experienced in form of goosebumps and/or a shudder or shiver that spreads from the head to the back and/or other parts of the body and may be accompanied by intensely experienced emotions such as joy or sadness. These reactions occur in relation to different experiences such as contemplation of art or listening to music.

Please state on the horizontal lines below how often and how intensely you have experienced chills in response to the music that you have listened to at today’s appointment.

| Please state *how often* you experienced chills today while listening to the music. | | | | | | | | |
| --- | --- | --- | --- | --- | --- | --- | --- | --- |
| not at all |  |  |  |  |  |  |  | almost always |
| If you experienced chills today, please indicate *how intense* your chills were. | | | | | | | | |
| barely   noticeable |  |  |  |  |  |  |  | overwhelmingly strong |

# **QUESTIONNAIRE 2** Musical engagement between music-listening sessions

**Music-related experiences between appointments**

Please tick as appropriate.

1. How often have you listened to music since the last appointment?

never 1 2 3 4 5 very often

What does this mean for you (how many minutes overall)? _____________________

1. How often have you made music (e.g., singing) since the last appointment?

never 1 2 3 4 5 very often

What does this mean for you (how many minutes overall)? _____________________

**References**

1. Schulz P, Schlotz W, Becker P*. Trierer Inventar zum chronischen Stress (TICS).* Göttingen: Hogrefe (2004).
2. Schlotz W, Yim IS, Zoccola PM, Jansen L, Schulz P. The Perceived Stress Reactivity Scale: Measurement invariance, stability, and validity in three countries. *Psychol Assess.* (2011) 23(1):80–94. doi:10.1037/a0021148
3. Wilhelm P, Schoebi D. Assessing Mood in Daily Life. *European Journal of Psychological Assessment* (2007) 23(4):258–67. doi:10.1027/1015-5759.23.4.258
4. Nater UM, Krebs M, Ehlert U. Sensation Seeking, Music Preference, and Psychophysiological Reactivity to Music. *Musicae Scientiae* (2005) 9(2):239–54. doi:10.1177/102986490500900205
5. Linnemann A, Kreutz G, Gollwitzer M, Nater UM. Validation of the German Version of the Music-Empathizing-Music-Systemizing (MEMS) Inventory (Short Version). *Frontiers in Behavioral Neuroscience* (2018) 12. doi:10.3389/fnbeh.2018.00153
6. Saarikallio SH. Music in Mood Regulation: Initial Scale Development. *Musicae Scientiae* (2008) 12(2):291–309. doi:10.1177/102986490801200206
7. Abler B, Kessler H. Emotion Regulation Questionnaire – Eine deutschsprachige Fassung des ERQ von Gross und John. *Diagnostica* (2009) 55(3):144–52. doi:10.1026/0012-1924.55.3.144
8. Smets EM, Garssen B, Bonke B, Haes JC de. The Multidimensional Fatigue Inventory (MFI) psychometric qualities of an instrument to assess fatigue*. J Psychosom Res.* (1995) 39(3):315–25.
9. Buysse DJ, Reynolds CF, Monk TH, Berman SR, Kupfer DJ. The Pittsburgh Sleep Quality Index: A new instrument for psychiatric practice and research. *Psychiatry Res.* (1989) 28(2):193–213.
10. Rammstedt B, John OP. Measuring personality in one minute or less: A 10-item short version of the Big Five Inventory in English and German. *Journal of Research in Personality* (2007) 41(1):203–12. doi:10.1016/j.jrp.2006.02.001
11. Schulz U, Schwarzer R. Soziale Unterstützung bei der Krankheitsbewältigung: Die Berliner Social Support Skalen (BSSS). *Diagnostica* (2003) 49(2):73–82. doi:10.1026//0012-1924.49.2.7
12. Hautzinger M, Keller F, Kühner C. *Beck Depressions-Inventar Revision (BDI-II) Revision. 2nd edition.* Frankfurt: Pearson Assessment (2009).
13. Löwe B, Spitzer RL, Zipfel S, Herzog W. *Gesundheitsfragebogen für Patienten (PHQ D). Komplettversion und Kurzform Testmappe mit Manual, Fragebögen, Schablonen.* Karlsruhe: Pfizer (2002)
14. Ditzen B, Nussbeck F, Drobnjak S, Spörri C, Wüest D, Ehlert U. Validierung eines deutschsprachigen DSM-IV-TR basierten Fragebogens zum prämenstruellen Syndrom. *Zeitschrift für Klinische Psychologie und Psychotherapie* (2011) 40(3):149–59. doi:10.1026/1616-3443/a000095
